# Addendum: Gosling, E.M., *et al.* The Status of Pet Rabbit Breeding and Online Sales in the UK: A Glimpse into an Otherwise Elusive Industry. *Animals* 2018, *8*, 199

**DOI:** 10.3390/ani8120238

**Published:** 2018-12-13

**Authors:** Emma M. Gosling, Jorge A. Vázquez-Diosdado, Naomi D. Harvey

**Affiliations:** 1 Politics and Society, Faculty of Humanities and Social Sciences, University of Winchester, Sparkford Road, Winchester SO22 4NR, Hampshire, UK; 2 School of Veterinary Medicine and Science, The University of Nottingham, Nottingham LE12 5RD, Leicestershire, UK; svzjv@exmail.nottingham.ac.uk (J.A.V.-D.); Naomi.Harvey@nottingham.ac.uk (N.D.H.)

The authors would like to add the following statement to the published article [1]. The Acknowledgements of the original paper should be updated to the following version:

“**Acknowledgements**: The authors would like to thank James Oxley and Clare Ellis for their support and comments in the early stages of planning this project, in addition to the RSPCA, RWAF, and everyone else who helped to advertise the rabbit breeder questionnaire. Thank you also to Peter Craigon for proof reading an early version of the manuscript. The authors would like to give credit to Dr. Andrew Knight, Dr. Steven McCulloch, and Dr. Kelly Gouveia from the University of Winchester for their supervision of the MSc project that inspired this paper”.

The manuscript will be updated, and the original will remain online on the article’s webpage. The authors would like to apologize for any inconvenience caused to the readers by these changes.
