# Supplementary material for: The Status of Pet Rabbit Breeding and Online Sales in the UK: A Glimpse into an Otherwise Elusive Industry
Source: Animals (Basel). 2018 Nov 6;8(11):199. doi: 10.3390/ani8110199 (PMC6262429; doi:10.3390/ani8110199)
Supplement: Supplementary file 1 [file animals-08-00199-s001.pdf]

# Supplementary Material:

# The Status of Pet Rabbit Breeding and Online Sales in the UK: A Glimpse into an Otherwise Elusive Industry


**Emma Gosling [1]*, Jorge A. Vázquez-Diosdado [2] and Naomi D. Harvey [2,]**

[1] University of Winchester, Winchester, Hampshire, SO22 4NR; emma.gurney@hotmail.co.uk (E.G)
[2] School of Veterinary Medicine and Science, The University of Nottingham, Leicestershire, LE12 5RD, UK; Naomi.Harvey@nottingham.ac.uk (N.D.H.); svzjv@exmail.nottingham.ac.uk (J.A.V-D)
* Correspondence: emma.gurney@hotmail.co.uk


**Supplementary Table S1.** Freedom of information (FOI) request key dates

| *Council Name* | *1st FOI Sent* | *1st Response Received* | *2nd FOI Sent* | *2nd Response Received* |
|---|---|---|---|---|
| *Boston Borough Council* | 12.05.2017 | 17.05.2017 | 25.05.2018 | 30.05.2018 |
| *Erewash Borough Council* | 12.05.2017 | 17.05.2017 | 25.05.2018 | 21.06.2018 |
| *Devon County Council* | 12.05.2017 | 16.05.2017 | - | - |
| *Broadland District Council* | 12.05.2017 | 12.06.2017 | 25.05.2018 | 04.06.2018 |
| *Hart District Council* | 12.05.2017 | 17.05.2017 | 25.05.2018 | 05.06.2018 |
| *Cherwell District Council* | 12.05.2017 | | 25.05.2018 | 06.06.2018 |
| *Aberdeen City Council* | 12.05.2017 | 22.05.2017 | 25.05.2018 | 22.06.2018 |
| *Chelmsford Borough Council* | 12.05.2017 | 12.06.2017 | 25.05.2018 | 21.06.2018 |
| *Eden District Council* | 12.05.2017 | 17.05.2017 | 25.05.2018 | 30.05.2018 |
| *Harborough District Council* | 12.05.2017 | 22.05.2017 | 25.05.2018 | 29.05.2018 |
| *Caerphilly County Borough Council* | 12.05.2017 | 09.06.2017 | 25.05.2018 | 31.05.2018 |
| *Havant Borough Council* | 12.05.2017 | 16.05.2017 | 25.05.2018 | 25.05.2018 |
| *Aylesbury Vale District Council* | 12.05.2017 | 18.05.2017 | 25.05.2018 | 16.06.2018 |

| | | | | |
|---|---|---|---|---|
| *Clackmannashire Council* | 12.05.2017 | 17.05.2017 | 25.05.2018 | 08.06.2018 |
| *Derbyshire Dales District Council* | 12.05.2017 | 09.06.2017 | 25.05.2018 | 04.07.2018 |
| *Halton Borough Council* | 12.05.2017 | 18.05.2017 | 25.05.2018 | 04.06.2018 |
| *Fenland District Council* | 12.05.2017 | 23.05.2017 | 25.05.2018 | 01.06.2018 |
| *Derby City Council* | 12.05.2017 | 17.05.2017 | 25.05.2018 | 05.06.2018 |
| *Enfield Council* | 12.05.2017 | 30.05.2017 | 25.05.2018 | 29.05.2018 |
| *Arun Council* | 12.05.2017 | - | 25.05.2018 | 01.06.2018 |
| *Cheshire West and Chester* | 12.05.2017 | 05.06.2017 | 25.05.2018 | 14.06.2018 |
| *Dorset County Council* | 12.05.2017 | 15.05.2017 | 25.05.2018 | 29.05.2018 |
| *Darlington Borough Council* | 12.05.2017 | 26.07.2017 | 25.05.2018 | 11.06.2018 |
| *Doncaster council* | 12.05.2017 | 25.05.2017 | 25.05.2018 | 27.06.2018 |
| *Greenwich* | 12.05.2017 | 17.05.2017 | 25.05.2018 | 31.05.2018 |
| *Denbighshire County Council* | 12.05.2017 | 23.05.2017 | 25.05.2018 | 30.05.2018 |
| *Bury Metropolitan Borough Council* | 12.05.2017 | 23.05.2017 | 25.05.2018 | 20.06.2018 |
| *Christchurch Borough Council* | 23.07.2017 | 03.08.2017 | 25.05.2018 | |
| *Cornwall Council* | 12.05.2017 | 08.06.2017 | 25.05.2018 | 14.06.2018 |
| *Harrogate Borough Council* | 12.05.2017 | 21.06.2017 | 25.05.2018 | 05.07.2018 |
| *Harlow District Council* | 12.05.2017 | - | 25.05.2018 | 05.06.2018 |
| *East Hampshire District Council* | 12.05.2017 | 15.05.2017 | 25.05.2018 | 25.05.2018 |
| *Aberdeenshire Council* | 12.05.2017 | 22.05.2017 | 25.05.2018 | 29.05.2018 |

| | | | | |
|---|---|---|---|---|
| *Amber Valley Council* | 12.05.2017 | 15.05.2017 | 25.05.2018 | 04.07.2018 |
| *Fife Council* | 12.05.2017 | 16.05.2017 | 25.05.2018 | 14.06.2018 |
| *East Ayrshire Council* | 12.05.2017 | 12.06.2017 | 25.05.2018 | 28.05.2018 |
| *Croydon* | 12.05.2017 | 25.05.2017 | 25.05.2018 | 01.06.2018 |
| *Coventry City Council* | 12.05.2017 | 06.06.2017 | 25.05.2018 | 13.06.2018 |
| *Bromsgrove District Council* | 12.05.2017 | 26.06.2017 | 25.05.2018 | 04.07.2018 |
| *Great Yarmouth Borough Council* | 12.05.2017 | 06.07.2017 | 25.05.2018 | 08.06.2018 |

**Supplementary Figure S1**. Pet Shop licence application from Amber Valley Borough Council 2016

Amber Valley Borough Council

**PET ANIMALS ACT 1951**
**Application for Licence to keep a Pet Shop**

I/We [illegible]

Of (Home Address) [illegible]

Contact Telephone Number [illegible] E mail [illegible]

As [proposed] occupier(s) of the premises hereinafter mentioned HEREBY MAKE APPLICATION for a LICENCE TO KEEP A PET SHOP at the premises,

Address [illegible]

I/WE enclose herewith the sum of £100.00 being the amount of the fee payable on the Licence for which application is made and declare that the information provided in this form is correct.

Dated 21 DECEMBER 2016 Signed [illegible]

**Where do you normally display your Pet Shop licence?** ON A BOARD UNDER CARPORT

**PARTICULARS**
**Please outline below the species of animal are you wishing to sell in the forthcoming year.**

| Type of animals | Proposed numbers | Details of accommodation | Age at which proposed to be sold | What feed is provided to the animals? |
|---|---|---|---|---|
| Parrots | | | | |
| Pigeons | | | | |
| Other large birds | | | | |
| Budgerigars, finches and other small birds | | | | |
| Dogs | | | | |
| Cats | | | | |
| Rabbits and cavies | 40 | HUTCHES OUTSIDE FOR RABBIT INSIDE FOR GUINEA PIGS PLUS EXERCISE RUNS WHEN WEATHER PERMITS | 8 WEEKS | DRY FOOD FRESH VEG HAY. |
| Hamsters | | | | |
| Rats, mice and gerbils | | | | |
| Tortoises | | | | |

N:\Regulation\04 Animal Welfare (Licensing)\Application Letters And Forms\App For Pet Shop Renewal.Doc

**Supplementary Figure S2**. Pet shop licence application from East Hampshire District Council 2017

East Hampshire DISTRICT COUNCIL

SCANNED

ATTACHED

CK/ /17

**PET ANIMALS ACT 1951**
**APPLICATION FOR LICENCE TO KEEP A PET SHOP**

**I/WE** [illegible] ..............................

**Of** [illegible] ..............................

As [proposed] occupier(s) of the premises hereinafter mentioned **HEREBY MAKE APPLICATION** in pursuance of section 1 of the Pet Animals Act 1951, for a **LICENCE TO KEEP A PET SHOP** at the premises of which particulars are given below.

| | |
|---|---|
| Postal address of premises and telephone number: | [illegible] |
| Address of Head Office, if Limited Company: | n/A |
| Number and size of rooms in which business will be carried on: | In garden and Bunny Barn. (10×16) feet garden is 50 feet by 65 approx |
| Heating arrangements: | Barn has a gas heater for winter. doors open / air / ventilation in roof |
| Method of ventilation of premises: | outdoor accommodation - Barn - open during day, double doors open |
| Lighting arrangements (natural and artificial): | all natural light - fairy lights in barn at night |
| Water supply: | Mains water |
| Arrangements for food storage: | In barn and in house. Hay bales in covered area at side of house. |
| Arrangements for disposal excreta: | collected weekly and taken to tip, is also made into compost. |

1

**Supplementary Table S2.** Frequency and proportion of rabbit breeds in one month of Pets4Homes sale adverts

| *Breed* | *Adverts* | *% of adverts* |
|---|---|---|
| *Mini Lop* | 1355 | 39.3% |
| *Mixed Breed* | 499 | 14.5% |
| *Netherland Dwarf* | 477 | 13.8% |
| *Lionhead* | 335 | 9.7% |
| *Mini Lion Lop* | 167 | 4.8% |
| *Rex* | 158 | 4.6% |
| *French Lop* | 113 | 3.3% |
| *Continental Giant* | 70 | 2.0% |
| *Dwarf Lop* | 56 | 1.6% |
| *Dutch* | 55 | 1.6% |
| *English* | 31 | 0.9% |
| *Angora* | 17 | 0.5% |
| *English Spot* | 14 | 0.4% |
| *Belgian Hares* | 13 | 0.4% |
| *English Lop* | 13 | 0.4% |
| *British Giant* | 10 | 0.3% |
| *New Zealand* | 10 | 0.3% |
| *Giant Papillon* | 9 | 0.3% |
| *German Lop* | 8 | 0.2% |
| *Californian* | 6 | 0.2% |
| *Harlequin* | 5 | 0.1% |
| *Argente* | 3 | 0.1% |
| *Himalayan* | 3 | 0.1% |
| *Cashmere Lop* | 2 | 0.1% |
| *Chinchilla* | 2 | 0.1% |
| *Dwarf Hotot* | 2 | 0.1% |
| *Golden Glavcot* | 2 | 0.1% |
| *Havana* | 2 | 0.1% |
| *Sable* | 2 | 0.1% |
| *Beveran* | 1 | 0.0% |
| *Flemish* | 1 | 0.0% |
| *Polish* | 1 | 0.0% |
| *Swiss Fox* | 1 | 0.0% |
| *Tan* | 1 | 0.0% |
| *Tri Coloured Dutch* | 1 | 0.0% |
| *Vienna* | 1 | 0.0% |
| ***Grand Total*** | **3446** | |

**Supplementary Table S3.** Types of rabbit breeds kept by questionnaire respondents (n=33 breeders)

| ***Breed*** | ***Number of breeders*** |
|---|---|
| *Mini Lop* | 21 |
| *Multiple Types* | 11 |
| *Netherland Dwarf* | 6 |
| *Lion head* | 6 |
| *Dutch* | 3 |
| *Mini Rex* | 3 |
| *French Lop* | 2 |
| *Lion Lop* | 2 |
| *Giants* | 1 |
| *All types* | 1 |
| *Lops* | 1 |
| *Dwarf Lop* | 1 |
| *Belgian Hares* | 1 |
| *Mini Plush Lop* | 1 |
| *Mini Dutch Lop* | 1 |
| *New Zealand* | 1 |
| *Silver Fox* | 1 |
